# *In Silico* Design and Biological Evaluation of a Dual Specificity Kinase Inhibitor Targeting Cell Cycle Progression and Angiogenesis

**DOI:** 10.1371/journal.pone.0110997

**Published:** 2014-11-13

**Authors:** Antony M. Latham, Jayakanth Kankanala, Gareth W. Fearnley, Matthew C. Gage, Mark T. Kearney, Shervanthi Homer-Vanniasinkam, Stephen B. Wheatcroft, Colin W. G. Fishwick, Sreenivasan Ponnambalam

**Affiliations:** 1 Endothelial Cell Biology Unit, School of Molecular & Cellular Biology, University of Leeds, Leeds LS2 9JT, United Kingdom; 2 School of Chemistry, University of Leeds, Leeds, LS2 9JT, United Kingdom; 3 Leeds Institute of Cardiovascular & Metabolic Medicine, Faculty of Medicine & Health, University of Leeds, Leeds LS2 9JT, United Kingdom; University of Bari Medical School, Italy

## Abstract

**Background:**

Protein kinases play a central role in tumor progression, regulating fundamental processes such as angiogenesis, proliferation and metastasis. Such enzymes are an increasingly important class of drug target with small molecule kinase inhibitors being a major focus in drug development. However, balancing drug specificity and efficacy is problematic with off-target effects and toxicity issues.

**Methodology:**

We have utilized a rational *in silico*-based approach to demonstrate the design and study of a novel compound that acts as a dual inhibitor of vascular endothelial growth factor receptor 2 (VEGFR2) and cyclin-dependent kinase 1 (CDK1). This compound acts by simultaneously inhibiting pro-angiogenic signal transduction and cell cycle progression in primary endothelial cells. JK-31 displays potent *in vitro* activity against recombinant VEGFR2 and CDK1/cyclin B proteins comparable to previously characterized inhibitors. Dual inhibition of the vascular endothelial growth factor A (VEGF-A)-mediated signaling response and CDK1-mediated mitotic entry elicits anti-angiogenic activity both in an endothelial-fibroblast co-culture model and a murine *ex vivo* model of angiogenesis.

**Conclusions:**

We deduce that JK-31 reduces the growth of both human endothelial cells and human breast cancer cells *in vitro*. This novel synthetic molecule has broad implications for development of similar multi-kinase inhibitors with anti-angiogenic and anti-cancer properties. *In silico* design is an attractive and innovative method to aid such drug discovery.

## Introduction

Vasculogenesis is the *de novo* formation of a vascular network whereas angiogenesis is the sprouting of new blood vessels [1], [2]. However, angiogenesis is subverted in pathophysiological conditions such as tumor growth and metastasis [2]. Vascular endothelial growth factor receptor 2 (VEGFR2) is a transmembrane receptor tyrosine kinase expressed by vascular endothelial cells and is essential for both vasculogenesis and angiogenesis [3]. VEGFR2 is also expressed in other vascular cell types and neurons but the functional significance is unclear [4], [5]. Binding of vascular endothelial growth factor A (VEGF-A) to VEGFR2 promotes receptor dimerization and tyrosine autophosphorylation, leading to activation of signaling molecules including phospholipase Cγ1 (PLCγ1), Akt and extracellular signal-regulated kinase (ERK1/2) [3]. Functional outputs of the VEGF-A-VEGFR2 axis include endothelial cell migration, enhanced cell survival and ultimately morphogenesis of hollow tubular conduits [6], [7]. In addition, uncontrolled cell division is a classic hallmark of solid tumor development and metastasis [8]. Cyclin-dependent kinase 1 (CDK1; cdc2) is a serine/threonine protein kinase required for mitotic progression in human cells [9] and heterodimerization with a regulatory cyclin subunit controls serine/threonine protein kinase activity and targeting [10]. In particular, CDK1/cyclin B activity regulates cell cycle progression from G_2_ to M phase and is needed to complete mitosis [11], [12].

The development of kinase inhibitors with increased target specificity often comes at the cost of reduced efficacy, principally due to redundancy in signaling pathways [13]. Thus the rational design of so-called ‘selectively non-selective’ inhibitors that target a unique and distinct subset of kinases is emerging as an attractive strategy for disease therapy [13]–[15]. To this end, we hypothesized that a dual small molecule inhibitor that simultaneously targets the VEGFR2 tyrosine kinase and CDK1 serine/threonine kinase would perturb both new blood vessel growth and cell cycle progression by targeting endothelial cells and cancer cells simultaneously. In this study, we used a combination of cutting-edge *in silico* modeling approaches and informed design to synthesize a novel aminotriazole-based compound (termed JK-31), which was predicted to bind to the active sites of both the VEGFR2 and CDK1 protein kinases. Here we show that such dual inhibition of VEGFR2 and CDK1 kinase activity perturbs proliferation of breast cancer cells and is a potent anti-angiogenic agent, both *in vitro* and in an *ex vivo* murine model.

## Materials and Methods

### Ethics statement

Mouse studies were carried out in accordance with institutional and national regulatory procedures under an animal project licence approved by the UK Home Office.

### Chemicals

Chemicals were purchased from Sigma-Aldrich (Poole, UK), Merck (Nottingham, UK) or VWR (Lutterworth, UK) unless otherwise stated.

### Cell culture, pharmacology and immunoblotting

Human primary endothelial cells were isolated, cultured and validated for classical endothelial markers (Figure S1A-S1E) as previously described [16], [17]. All experiments were conducted at <6 cell passages. For growth factor stimulation studies, cells were serum-starved overnight in MCDB-131 (Invitrogen) containing 0.2% (w/v) bovine serum albumin (BSA), pre-treated with JK-31 for 30 min and stimulated as described in accompanying figure legends. Recombinant human VEGF-A_165_ was a gift from Genentech Inc. (San Francisco, CA, USA), recombinant human basic fibroblast growth factor (bFGF) and epidermal growth factor (EGF) were purchased from R&D Systems (Abingdon, UK), acidic FGF (aFGF) was a gift from ImmunoTools (Friesoythe, Germany) and insulin-like growth factor-1 (IGF-1) was a gift from Hema Viswambharan (University of Leeds, UK). Human MCF-7 breast cancer epithelial cells were from Cancer Research UK (London, UK) and were cultured in Dulbecco's modified eagle medium (DMEM) containing 10% (v/v) fetal bovine serum (FBS). Vatalanib was from Novartis AG (Basel, Switzerland), sunitinib was from Enzo Lifesciences (Exeter, UK) and staurosporine was provided by Reaction Biology Corp. (Malvern, USA). SDS-PAGE electrophoresis and immunoblotting were carried out as previously described [18]. Antibodies to cyclin A, cyclin B, cyclin D1 and CDK1 were from BD Transduction Labs (Oxford, UK). Antibodies to FGFR1 and PLCγ1 were from Santa Cruz Biotechnology (CA, USA). Also used were anti-VEGFR2 extracellular domain antibody (R&D Systems), anti-α-tubulin antibody (Sigma-Aldrich) and anti-β-actin antibody (AbCam, Cambridge, UK). All other antibodies were from Cell Signalling Technologies (Danvers, USA).

### Chemical synthesis of JK-31

To a solution of N-cyano-S-methyl-N'phenylisothiourea (0.20 g, 1.04 mmol, 1.0 eq) in ethanol (10 ml), hydrazine hydrate (50 µl, 1.04 mmol, 1.0 eq) was added and heated to reflux until the starting material was consumed. The solvent was evaporated *in vacuo* and triturated with ether to leave N-phenyl-1*H*-1,2,4-triazole-3,5-diamine (JK-31/INT), a colorless solid (0.16 g, 0.88 mmol, 85%). To a solution of JK-31/INT (0.10 g, 0.57 mmol, 1.0 eq) in 5 ml acetone at 0°C, pyridine (50 µl, 0.57 mmol, 1.0 eq) and 4- tert-butyl-benzoyl chloride (100 µl, 0.57 mmol, 1.0 eq) was added. The reaction mixture was stirred at room temperature for 4 h. The solvent was removed under reduced pressure and the residue diluted in water (10 ml). Solids were collected by filtration, washing with water, and the purified by flash chromatography (95∶5 CH_2_Cl_2_/CH_3_OH) to afford (5-amino-3-(phenylamino)-1H-1,2,4-triazol-1-yl)(4-(tert-butyl)phenyl)methanone (JK-31) as a colorless solid (0.11 g, 0.31 mmol, 56%).

### Nuclear magnetic resonance (NMR) spectroscopy

Proton nuclear magnetic resonance (^1^H NMR) spectra were recorded at 500 MHz on a Bruker DRX500 instrument (Coventry, UK) as solutions in deuterated dimethyl sulfoxide (DMSO). Chemical shifts are given in parts per million (ppm) with reference to TMS in DMSO (2.5 ppm).^13^C NMR spectra were recorded at 125 MHz. Chemical shifts were quoted in ppm with reference to the central peak of the deuterated DMSO at 40 ppm. Coupling constants (J) were quoted in Hertz (Hz). Multiplicities were given as singlet (s), doublet (d), triplet (t). Electrospray (ES) spectra were recorded in house on a Bruker Daltonics micrOTOF spectrometer. Thin layer chromatography (TLC) was performed using pre-coated glass backed silica gel 60 F254 plates (Merck). The plates were visualized using a ultraviolet (UV) lamp or by dipping in a solution of permanganate. Silica gel 60 (particle size 37–70 µm; Merck) was used for flash chromatography. HPLC analyses were carried out on Dionex HPLC system using a Hyperprep HS C18 column with a gradient of acetonitrile and water (5–95%) with 0.1% TFA at a flow rate of 0.5 ml/min over a period of 5 min.

Spectra for JK-31: ^1^H NMR (500 MHz, DMSO d_6_): 9.27 (s, 1H), 8.16 (d, J 8.5 Hz, 2H), 7.82 (br s, 2H), 7.60 (d, J = 8.5 Hz, 2H), 7.54 (d, J 7.5 Hz, 2H), 7.23 (t, J 7.5 Hz, 2H), 6.85 (t, J = 7.5 Hz, 1H), 1.33 (s, 9H); ^13^C NMR (125 MHz, DMSO d_6_): 166.3, 158.2, 157.4, 155.6, 140.9, 130.4, 129.7, 128.6, 124.7, 120.0, 116.6, 34.8, 30.8; Mass (ES+): C_19_H_21_N_5_O requires 335.1746, Found: 358.1638 (M+Na), HPLC (R_T_): 3.88 mins (100%), CHN analysis (%): C_19_H_21_N_5_O requires C: 68.04, H: 6.31, N: 20.88 Found: C: 67.70, H: 6.20, N: 20.71.

### Computational docking studies, informed molecular design and homology modelling

As described above, JK-31 was conceived by simultaneous analysis of the structure-activity relationship of known CDK1 and VEGFR2 inhibitors. For example with reference to the CDK1 inhibitor JNJ-7706621, JK-31 contains the same aminotriazole core, but the removal of a sulphonamide moiety and replacement of a 2,6-difluorophenyl group with a 4-(tert-butyl)phenyl group was predicted to aid binding to VEGFR2 whilst retaining CDK1-inhibiting properties. *Tert*-butyl groups are observed in a number of small molecule receptor tyrosine kinase inhibitors e.g. PD173074 (Figure S2A). The binding pose of JK-31 was validated against a number of known VEGFR2 and CDK1 inhibitors *in silico* (Figure S3 and S4). The diaminothiazole compound shown in Figure S2B and Figure S2C shares structural similarities with JNJ-7706621.

**Figure 1:**
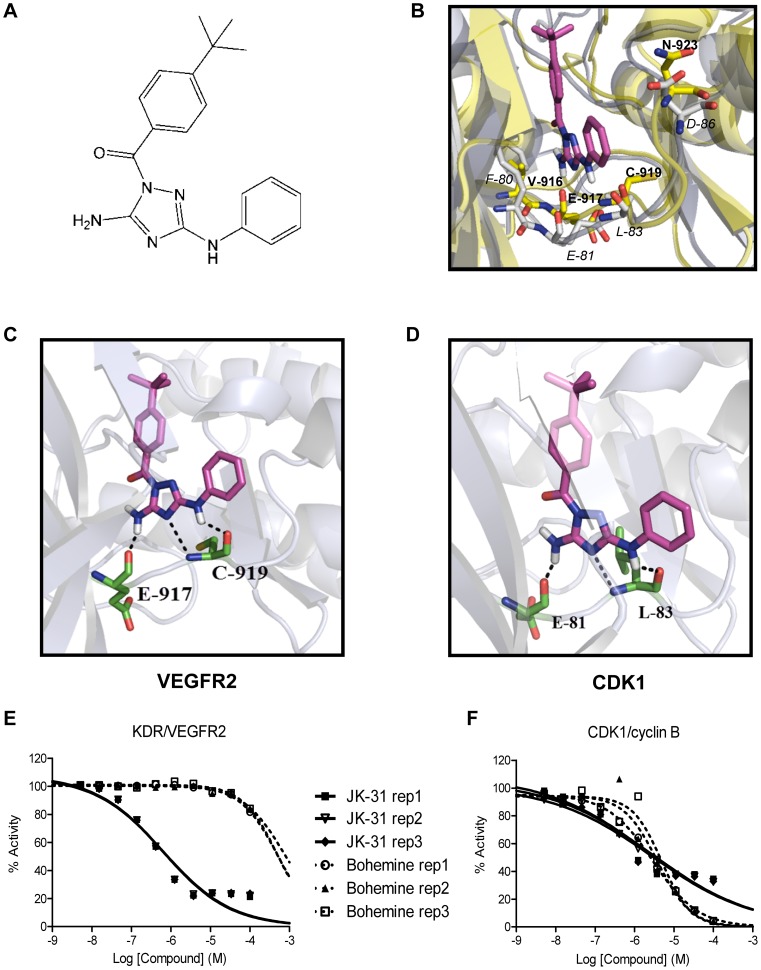
Dual targeting of VEGFR2 and CDK1. (A) Chemical structure of the aminotriazole-based compound JK-31. A full description of the chemical synthesis can be found in the Materials and Methods section. (B) *In silico* molecular modeling of JK-31 (magenta structure) in the VEGFR2 and CDK1 kinase domains. A homology model of CDK1 was created using a structurally-related family member (CDK2 PDB code: 3s2p). The CDK1 and VEGFR2 kinase domain sequences were aligned and overlapped. JK-31 was docked into the overlapped crystal structure using Glide program and important amino acid residues identified. VEGFR2 crystal structure is shown in yellow and residues annotated in bold; CDK1 crystal structure is shown in blue/grey and residues annotated in italics. (C) JK-31 was docked into a VEGFR2 crystal structure alone (PDB code: 3cjg) and predicted hydrogen bond contacts identified. (D) JK-31 was docked into the CDK1 crystal structure homolog alone and predicted hydrogen bond contacts identified. Dotted lines denote predicted hydrogen bond contacts. Green structures denote important kinase domain residues. Further details of these models can be found in Figure S2. (E–F) JK-31 inhibits the intrinsic kinase activity of both CDK1 and VEGFR2. IC_50_ curves were generated by incubating recombinant protein kinases with a peptide substrate, radiolabeled [γ-^33^P]-ATP and either JK-31 or bohemine (5 nM to 100 µM). Effects of JK-31 and bohemine on phosphate transfer from a (E) recombinant human VEGFR2 to a peptide substrate, poly[Glu∶Tyr] (4∶1) and (F) recombinant human CDK1/cyclin B complex to histone H1 substrate *in vitro*. Line graphs of three independent replicate experiments are shown.

Due to the high sequence homology between CDK1 and CDK2 (66% homology in kinase domain), an available crystal structure of CDK2 (PDB code: 3s2p) [19] was used to build a homology model of CDK1. The sequence of CDK1 was imported into the program Prime (Schrödinger Inc.) [20] and the model of CDK1 was built using 3s2p as a template, which was further subjected to protein preparation using Maestro and PrimeX (Schrödinger Inc.) (Figure 1B, Figure S2) [21]. Use of the programs SPROUT (SimBioSys Inc.) [22], [23] and Glide (Schrödinger Inc.) [24]–[26] for molecular modelling have been described previously [27]. Briefly, JK-31 was docked into both the protein kinase domains of either CDK1 and VEGFR2 (PDB code: 3cjg) [28] using both programs. SPROUT identified a target region where JK-31 would interact most strongly and was scored to give an estimated pK_i_ (Figure 1B, Table 1). Glide was also used to predict the binding affinity of JK-31 (presented as a Glide score where a lower score represents lower energy and thus greater affinity; Table 1). Images from Glide software are used in this publication (Figures 1B-1D, S2A and S2B). The binding mode of JK-31 within the VEGFR2 and CDK1 kinase domains (with respect to hydrogen bonding) were confirmed to be similar to oneanother (Figures 1B–1D). A full description of the structure-activity relationship of JK-31 and other compounds of the same class is currently ongoing.

**Table 1:** Estimated binding affinities (pK_i_ values) of JK-31 to VEGFR2 and CDK1 kinase domains *in silico*, as predicted using SPROUT and Glide programs (see Methods).

	JK-31	
	Estimated pK_i_ (SPROUT)	Estimated pK_i_ (Glide)
**VEGFR2**	−9.19	−9.75
**CDK1**	−8.77	−7.13

### *In vitro* kinase profiling assay

JK-31, bohemine, vatalanib and staurosporine were screened for inhibitory activity against the VEGFR2 and CDK1 kinases using a ^33^P receptor tyrosine *in vitro* kinase HotSpot^SM^ profiling assay (Reaction Biology, Malvern, USA). Ten-point IC_50_ profiles were generated using concentrations of JK-31 between 5 nM and 100 µM and a [γ-^33^P]-ATP concentration of 10 µM.

### Animal studies and *ex vivo* murine aortic ring angiogenesis assay

Three male five-month old wild type C57Bl/6 mice were humanely sacrificed followed by cervical dislocation. A thoraco-abdominal incision was made and the heart and aorta excised and stored in Hank's balanced buffer. The aortas were cleaned by removing connective tissue and adventitia from the exterior and each aorta was cut into 18–24 rings. Ring explants were embedded into growth-factor reduced Matrigel basement membrane matrix (Beckton Dickinson) in a 24-well plate and incubated for 45 min at 37°C to allow the Matrigel to set and the rings to adhere. Rings were cultured *ex vivo* in MV2 mouse endothelial cell growth medium (Promocell, Heidelberg, Germany) for six days in the presence or absence of inhibitors, replacing the medium every two days. Rings were photographed and the degree of sprouting measured using Cell∧B imaging software (Olympus, Tokyo, Japan). The mean of the three longest angiogenic sprouts on each ring was measured.

### Organotypic tubulogenesis assay

This assay was performed as previously described [18], [27]. Primary human foreskin fibroblasts (pHFF) were a gift from Gareth Howell (University of Leeds, UK) and were cultured routinely in DMEM containing 10% (v/v) FBS up to passage 10. In co-culture with HUVECs, the medium was replaced for endothelial cell growth medium (Promocell, Heidelberg, Germany).

### Cell proliferation and scratch wound healing assays

Cell proliferation was measured using a bromodeoxyuridine (BrdU) incorporation assays (Roche Diagnostics, Burgess Hill, UK), performed in a 96-well plate with 2000 cells/well as previously described [18], [27]. For BrdU assays, cells were treated with inhibitors for 16 h prior to assay. Scratch wound healing assays were performed as previously described [18]. Briefly, HUVECs were grown to confluence and starved for 3 h in serum-free MCDB131 medium (Invitrogen, Amsterdam, Netherlands) containing 0.2% (w/v) BSA. Cells were pre-treated with JK-31 for 1 h prior to making a vertical scratch wound through the cell monolayer with a sterile 200 µl plastic pipette tip of ∼0.9 mm tip width. Wounded cell monolayers were washed once with PBS and photographed. Cells were stimulated with 25 ng/ml VEGF-A for 16 h in the presence or absence of JK-31 and wounded cell monolayers photographed once more. Wound widths were measured using Image J software and % wound closure calculated by ((width before – width after)/width before) ×100.

### MTS cell viability assay

2000 endothelial cells were seeded per well of a 96-well plate and cultured in ECGM overnight. ECGM was then aspirated and cells starved in MCDB131 +0.2% (w/v) BSA for 2 h. After 2 h, media was changed for ECGM with or without DMSO, plus or minus 100 nM, 1 µM, 10 µM or 50 µM JK-31 and cells incubated for 48 h. Then, 10 µl of MTS reagent (CellTiter 96 AQueous Non-Radioactive Cell Proliferation Assay, Promega, Madison, Wisconsin, USA) was added to each well after 44 h. After further incubation for 4-6 h, we monitored the color change caused by reduction of the yellow tetrazolium compound (MTS) by metabolically active cells to brown formazan. Change in color was monitored at 490 nm using a Tecan Sunrise multiwavelength 96-well plate reader (Tecan, Mannedorf, Switzerland).

### Flow cytometry analysis of cell cycle progression

Endothelial (HUVECs) or human breast cancer (MCF-7) cells were cultured to subconfluency (60–70% confluent) and treated with either DMSO carrier alone or 200 nM nocodazole, 10 µM bohemine, 100 nM sunitinib or JK-31 (1, 10 or 50 µM) in DMSO for 48 h. Endothelial cells were detached from the tissue culture plastic using TrypLE Express (Invitrogen, Amsterdam, Netherlands) and quenched with DMEM containing 10% (v/v) FCS and cells transfered into 1.5 or 15 ml centrifuge tubes as appropriate. Cells were pelleted at 140 *g* at 4°C for 5 min and supernatant discarded. Cells were fixed in ice-cold 70% (v/v) ethanol added dropwise and stored at −20°C prior to analysis. Immediately before flow cytometry, cells were pelleted (140 *g* at 4°C for 5 min), supernatant removed and cells washed twice in 500 µl PBS. 100 µg/ml ribonuclease and 50 µg/ml propidium iodide (Sigma-Aldrich) was added to each sample and incubated for 2–3 h at 37°C. Cells were pelleted (140 *g* at 4°C for 5 min), supernatant removed and cells washed in 500 µl PBS before being resuspended in 500 µl PBS buffer containing 2.5 mM EDTA. Samples were gated and run at a low flow rate (<1000 events s^−1^) on a Fortessa flow cytometer (Becton Dickinson, Oxford, UK) with multi-laser and detection capabilities. The information from 10000 events was collect and data was analyzed using ModFit software (Becton Dickinson).

### Statistical analysis

Statistical analysis was performed using GraphPad Prism software (La Jolla, CA, USA). For comparison between more than two groups of data, one-way ANOVA with Tukey's post hoc test was used. Associated *p* values are noted in the figure legends. Error bars denote ± standard error of the mean (SEM).

## Results

### Design and *in silico* validation of JK-31: a dual VEGFR2/CDK1 inhibitor

We performed a simultaneous analysis of the structure-activity relationship of known CDK1 inhibitors e.g. AT7519 [29], [30] and JNJ-7706621 [31] and VEGFR2 inhibitors e.g. sunitinib, pazopanib, vatalanib and PD173074 [18], [27], [28], [32] to guide the design of a compound based on an aminotriazole pharmacophore. This molecule, JK-31, retains common structural features of both VEGFR2 and CDK1 inhibitors (see Experimental Section for further details of molecular design). Using the *in silico* molecular modeling programs SPROUT and Glide, JK-31 was predicted to bind with high affinity to the ATP-binding pocket of both the VEGFR2 and CDK1 kinase domains respectively (Figure 1 and Table 1).

We created a homology model of the CDK1 kinase domain using information from the crystal structure of a related kinase, CDK2 (PDB code: 3s2p) [19]–[21] (Figure S2B). The overall homology of the VEGFR2 and CDK1 kinase domains was relatively low (21.6%; Figure 1 and Figure S2A); however, structural similarities exist within the ATP-binding region (Figure 1B). In particular, using both programs SPROUT and Glide [22]–[26], JK-31 was predicted to make three hydrogen bond contacts with the VEGFR2 kinase domain (one with E917 and two with C919) and with homologous residues in the CDK1 kinase domain (one with E81 and two with L83) (Figures 1B–1D). The program SPROUT predicted JK-31 to bind to both VEGFR2 and CDK1 with a pK_i_ of -7 or less (corresponding to a predicted binding affinity in the nanomolar range, Table 1), with a slightly better affinity predicted in the case of VEGFR2 (Table 1). Further details of the docking studies are described in Figure S2. Validation studies of the CDK1 homology model using previously-characterized CDK1 inhibitors AT7519 [29], [30] and a diaminothizaole compound described by Schonbrunn and colleagues [33] are shown in Figure S3. Validation of the binding pose of JK-31 in the VEGFR2 kinase domain using previously-characterized VEGFR2 inhibitors PD173074 [32], a derivative of pazopanib and JK-P3 [27] is shown in Figure S4. JK-31 was synthesized from commercially available reagents as described in the Materials and Methods section.

### JK-31 inhibits the intrinsic protein kinase activity of both VEGFR2 and CDK1

To test the proposed ‘dual inhibitory’ potential of JK-31, we examined the ability of this molecule to inhibit the intrinsic kinase activities of either recombinant VEGFR2 or a recombinant CDK1/cyclin B heterodimer *in vitro* using a [γ-^33^P]-ATP kinase assay with specific peptide substrates (Table 2; Figures 1E and F). JK-31 showed dose-dependent inhibition of both protein kinases but as predicted, displayed more potent inhibition of the recombinant VEGFR2 receptor tyrosine kinase (Table 2; Figures 1E and 1F). Intriguingly, JK-31 displayed greater inhibition of CDK1 in comparison to a previously reported CDK1-specific inhibitor, bohemine (Table 2 and Figure 1F) and showed only slightly weaker inhibition of VEGFR2 in comparison to a VEGFR-selective inhibitor, vatalanib (Table 2).

**Table 2:** Inhibition of VEGFR2 and CDK1/cyclin B kinase activity by JK-31.

	VEGFR2		CDK1/cyclin B	
	*IC_50_ (µM)*	+ *SEM*	*IC_50_ (µM)*	+ *SEM*
**JK-31**	0.627	0.0107	2.54	0.194
**Bohemine**	0	0	3.89	0.594
**Vatalanib**	0.32	0.02	-	-
**Staurosporine**	0.00666	-	0.00167	-

IC_50_ values were derived from curves (Figures 1E and 1F) generated using an *in vitro* kinase assay (see Methods).

Another CDK1 inhibitor, JNJ-7706621, was shown to also inhibit VEGFR2 *in vitro* [31]. When directly comparing the IC_50_ values of JNJ-7706621 in a previous study with those of JK-31 in this study, JNJ-7706621 is more potent than JK-31 in terms of absolute IC_50_ values. However, when comparing relative potency, JNJ-7706621 is a∼17-fold more potent inhibitor of CDK1 than VEGFR2, whereas JK-31 is ∼4-fold more selective for VEGFR2 than CDK1. This exemplifies our success in engineering a compound with true dual targeting characteristics.

### JK-31 inhibits VEGF-A-stimulated signal transduction in primary human endothelial cells

**Figure 2:**
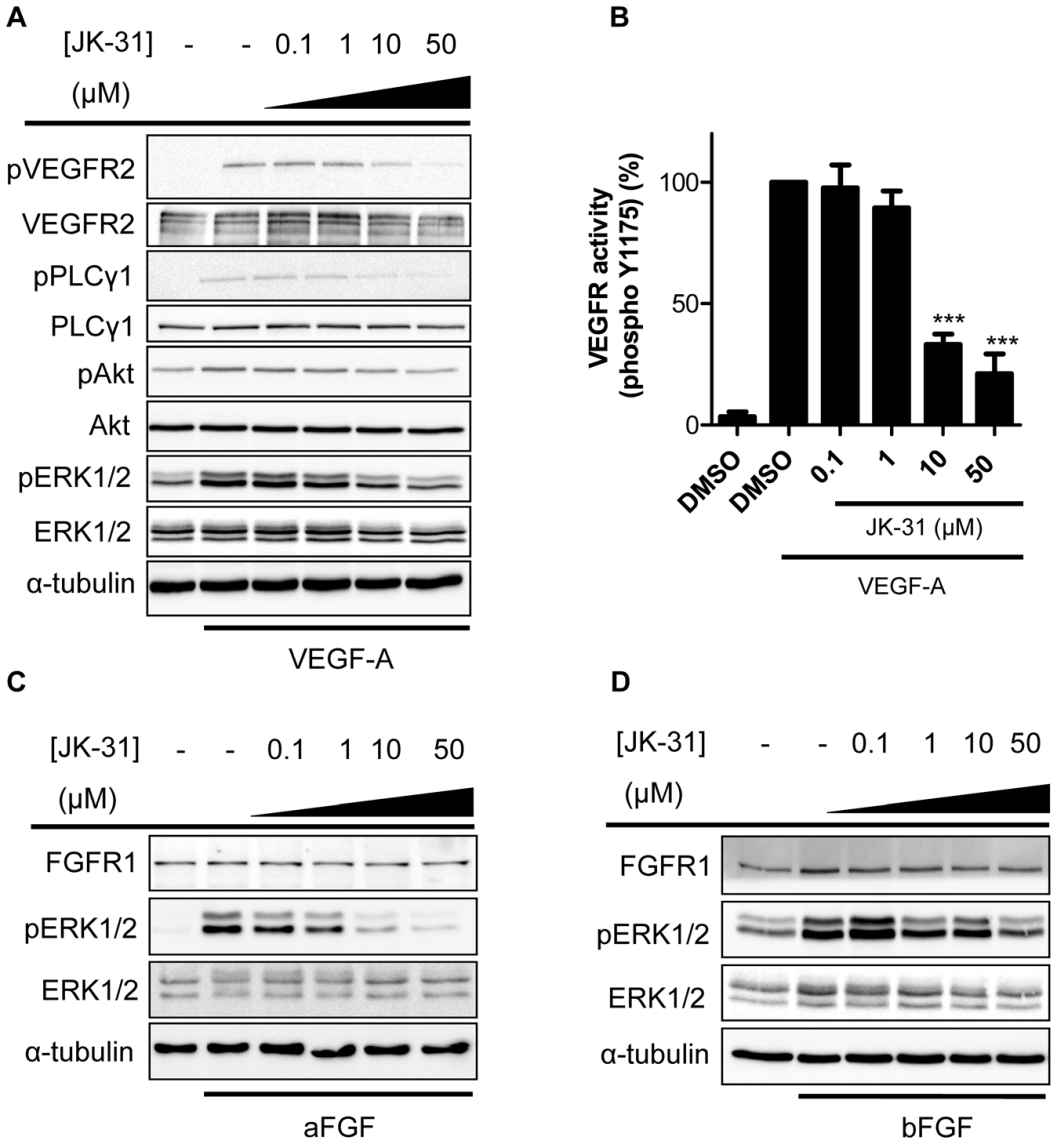
JK-31 inhibits growth factor-stimulated receptor activation and downstream signal transduction. (A) HUVECs were pre-treated with JK-31 (0, 0.1, 1, 10 or 50 µM) for 30 min followed by 7.5 min stimulation with VEGF-A (25 ng/ml) in the presence of JK-31. Total cell lysates were prepared and processed for immunoblotting. Levels of phosphorylated VEGFR2, PLCγ1, Akt and ERK were analysed using phospho-specific antibodies. Membranes were stripped and re-probed for total protein levels and a loading control (α-tubulin). (B) Densitometric quantification of phosphorylated VEGFR2-pY1175 levels in response to VEGF-A and JK-31 treatment. Error bars represent ± SEM (n = 4; ***p<0.001). (C-D) JK-31 inhibits FGF-stimulated intracellular signaling in endothelial cells. HUVECs were pre-treated with JK-31 (0, 0.1, 1, 10 or 50 µM) for 30 min followed by 10 min stimulation with either (C) bFGF (50 ng/ml) or (D) aFGF (50 ng/ml) in the presence of JK-31. Cells were lysed and processed for immunoblotting. Representative immunoblots of three independent experiments are shown.

A key feature of VEGFR kinase inhibitors is their ability to block VEGF-A-stimulated downstream signaling in endothelial cells [3], [6], [18], [27], [34]. Primary human umbilical vein endothelial cells (HUVECs) recapitulate many features of *in vivo* vascular function, including the expression of VEGFR2, Von Willebrand Factor (VWF) and PECAM-1 (Figure S1). We used immunoblot analysis of HUVECs treated with VEGF-A and JK-31 to examine the effects on VEGFR2 autophosphorylation and downstream signaling to PLCγ1, Akt and ERK1/2 (Figures 2A and B). At concentrations of 10 µM and above, JK-31 caused>60% inhibition of phosphorylation of residue Y1175 within the VEGFR2 cytoplasmic domain (Figure 2B). JK-31 also inhibited VEGF-A-stimulated signal transduction in a dose-dependent manner affecting multiple phosphorylation events such as production of PLCγ1-pY783, Akt-pS473 and ERK1/2-pT202/pY204 epitopes (Figure 2A). Quantification and statistical analysis clearly showed a dose-dependent inhibition of VEGF-A-stimulated signal transduction leading to altered phosphorylation status of key enzymes (Figure S5).

To assess the relative specificity of JK-31 for the VEGF-A-VEGFR2 pathway, we examined inhibition of signal transduction through additional growth factor receptor tyrosine kinase axes, namely acidic FGF (aFGF), basic FGF (bFGF), EGF and IGF-1 (Figures 2C and D; Figure S6A and S6B). JK-31 displayed dose-dependent inhibition of downstream ERK1/2 phosphorylation in response to either aFGF or bFGF (Figures 2C and D; Figure S5) but not in response to either EGF or IGF-1 (Figure S6A and S6B). Further work to fully characterize the selectivity profile of JK-31 against a wide range of targets using *in vitro* and cellular assays is currently ongoing.

### JK-31 has *in vitro* and *ex vivo* anti-angiogenic activity

**Figure 3:**
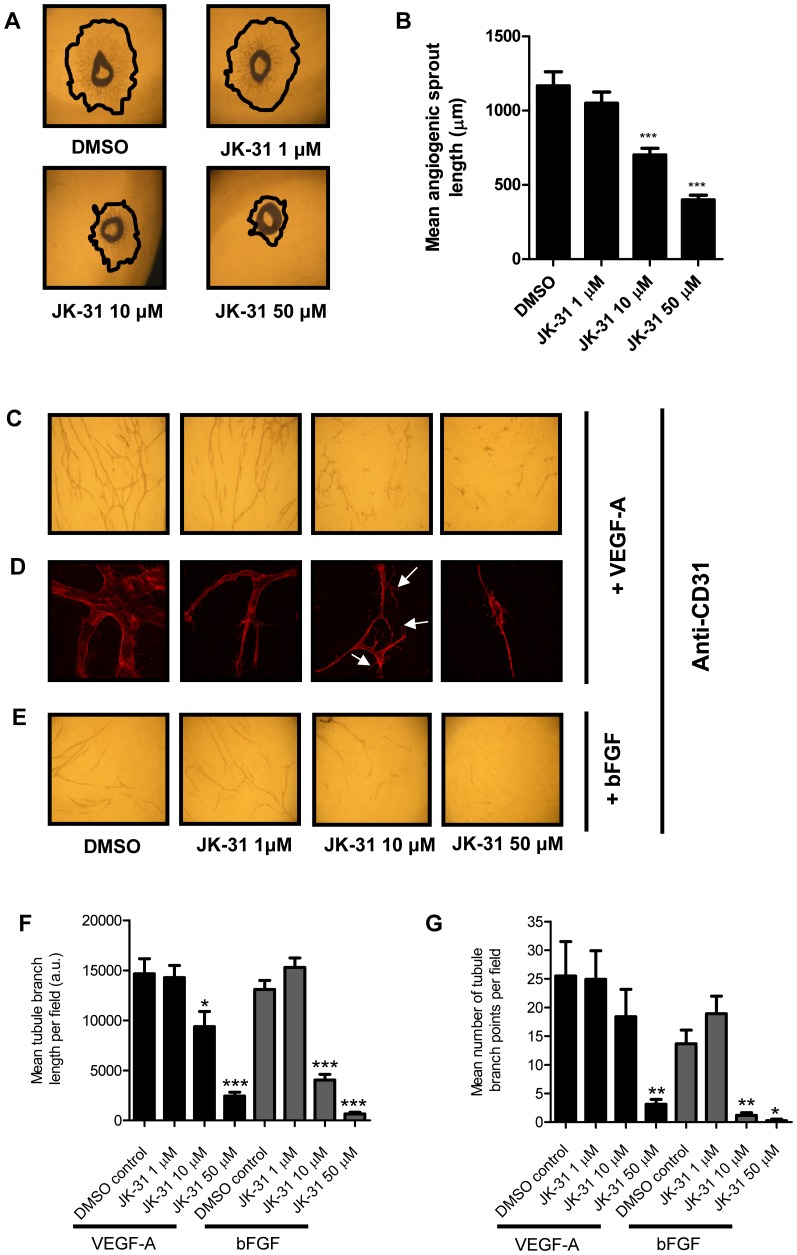
JK-31 inhibits angiogenesis in an *ex vivo* murine model and *in vitro*. (A) Effects of JK-31 on *ex vivo* angiogenic sprouting from wild-type mouse aortic ring explants. Aortic rings were seeded onto Matrigel and treated with JK-31 (0, 1, 10 or 50 µM) for six days and imaged by phase-contrast microscopy. Freehand black lines delineate the outermost extent of sprouting. (B) Absolute quantification of angiogenic sprout length. For each aortic ring, the mean of three longest sprouts was calculated. Error bars represent ±SEM (n = 18; ***p<0.001). (C) JK-31 inhibits endothelial tube formation *in vitro* in response to exogenous VEGF-A. HUVECs were seeded onto a confluent layer of primary fibroblasts and treated with VEGF-A (10 ng/ml) for seven days in the presence of DMSO or JK-31 (1, 10 or 50 µM). Co-cultures were stained with CD31 antibody followed by HRP-conjugated secondary antibody and visualized by light microscopy using 1,1-diaminobenzidine (DAB)-staining (see Materials and Methods). (D) Fluorescence microscopy analysis of endothelial cell phenotypes and filopodia formation during tubulogenesis in the presence of VEGF-A and JK-31. White arrowheads indicate vestigial lamellipodia-like structures. (E) JK-31 inhibits endothelial tube formation *in vitro* in response to exogenous bFGF (20 ng/ml). Treatment and processing was carried out as described above. Quantification of (F) mean tubule branch length and (G) mean number of tubule branch points in response to JK-31 and exogenous growth factors. Light micrograph fields were chosen at random and quantification performed using Image J software. Error bars represent ± SEM (n = 10; *p<0.05; **p<0.01; ***p<0.001) for each respective growth factor.

Multiple lines of evidence thus far in this study have revealed JK-31 as an inhibitor of pro-angiogenic receptor tyrosine kinase-regulated signal transduction in endothelial cells (Table 2; Figures 1E and 1F; Figure 2). Hence we assessed whether JK-31 could indeed inhibit angiogenesis using both *in vitro* and *ex vivo* models (Figure 3). In an *ex vivo* assay, mouse aortic ring explants were seeded onto Matrigel in full growth medium and incubated with VEGF-A and a dose range of JK-31 for six days (Figure 3A). At concentrations of 10 µM and above, JK-31 elicited more than 40% inhibition of radial angiogenic sprout formation (Figure 3B).

Secondly, an organotypic *in vitro* model of angiogenesis (where endothelial cells are grown on a confluent layer of fibroblasts) was used to assess the effects of JK-31 on VEGF-A-stimulated endothelial tubular morphogenesis (Figure 3C and 3D). *De novo* tubule formation and branching were examined both by conventional light microscopy (Figure 3C) and by high-resolution deconvolution microscopy (Figure 3D) using immunolabeling of the endothelial-specific protein CD31 (PECAM-1). JK-31 dose-dependently inhibited VEGF-A-stimulated endothelial tube formation (Figures 3C and 3D): approximately 35% inhibition was observed at 10 µM and more than 80% inhibition at 50 µM (Figure 3F).

Interestingly however, at 10 µM concentration of JK-31 and in the presence of VEGF-A, an aberrant hyper-branched endothelial tubule phenotype was observed with multiple vestigial lamellipodia-like structures emanating from the plasma membrane (Figure 3D, white arrowheads). This led to a higher branch count than predicted, despite reduced total CD31-positive staining (Figures 3F and 3G). At the highest concentration used (50 µM), more than 80% reduction in branching was observed (Figure 3G). Inhibition of bFGF-stimulated endothelial tube formation by JK-31 was more pronounced than for VEGF-A: ∼75% inhibition was observed at 10 µM and more than 95% inhibition at 50 µM (Figure 3E). In addition, bFGF-stimulated branching was more severely affected with more than 90% reduction at both 10 µM and 50 µM JK-31 (Figure 3G).

### JK-31 compromises endothelial wound healing *in vitro*

**Figure 4:**
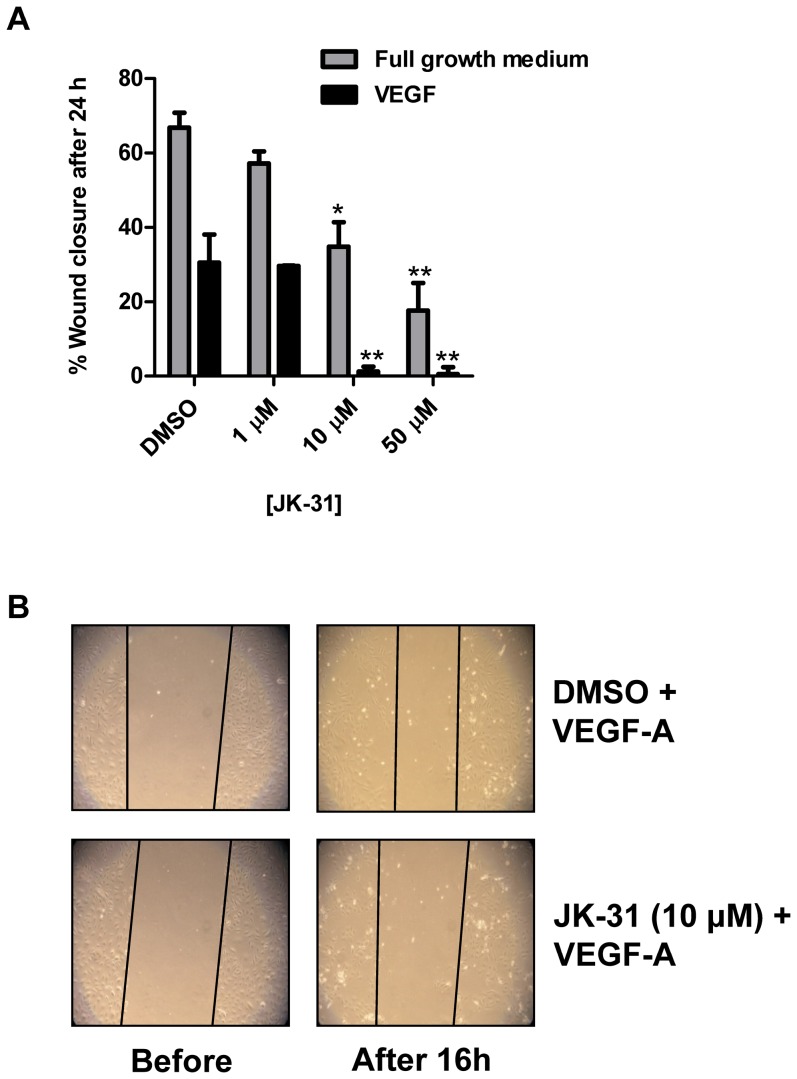
JK-31 inhibits endothelial wound closure *in vitro*. (A) Scratch-wounded endothelial cell monolayers were incubated in either full growth medium (grey bars) or stimulated with 25 ng/ml VEGF-A (black bars) for 16 h in the presence of DMSO or JK-31 (1, 10 or 50 µM). Wounded cell monolayers were photographed before and after treatment and wound widths at each time point were measured using Image J software. Percentage wound closure was calculated by [(width before – width after)/width before] ×100. Error bars represent ± SEM (n = 3; *p<0.05; **p<0.01) for each condition. (B) Representative phase-contrast micrographs depicting wound closure over 16 h in the presence VEGF-A and either DMSO or JK-31 (10 µM).

Endothelial cells in culture form a confluent cell monolayer [16]. A simple *in vitro* assay can be used to disrupt this cell monolayer, for example by a mechanical scratch wound, and the migration and proliferation of endothelial cells into the denuded region can be monitored over time. This model thus recapitulates early events during angiogenesis [6], [35]. We assessed the effects of JK-31 on endothelial cell monolayer wound closure during growth either in full growth medium or minimal medium supplemented with VEGF-A alone (Figure 4). At 10 µM concentration and above, JK-31 completely inhibited VEGF-A-stimulated wound closure (Figure 4A, 4B) and only partially inhibited wound closure in full growth medium (Figure 4A).

### JK-31 inhibition of human cell proliferation

**Figure 5:**
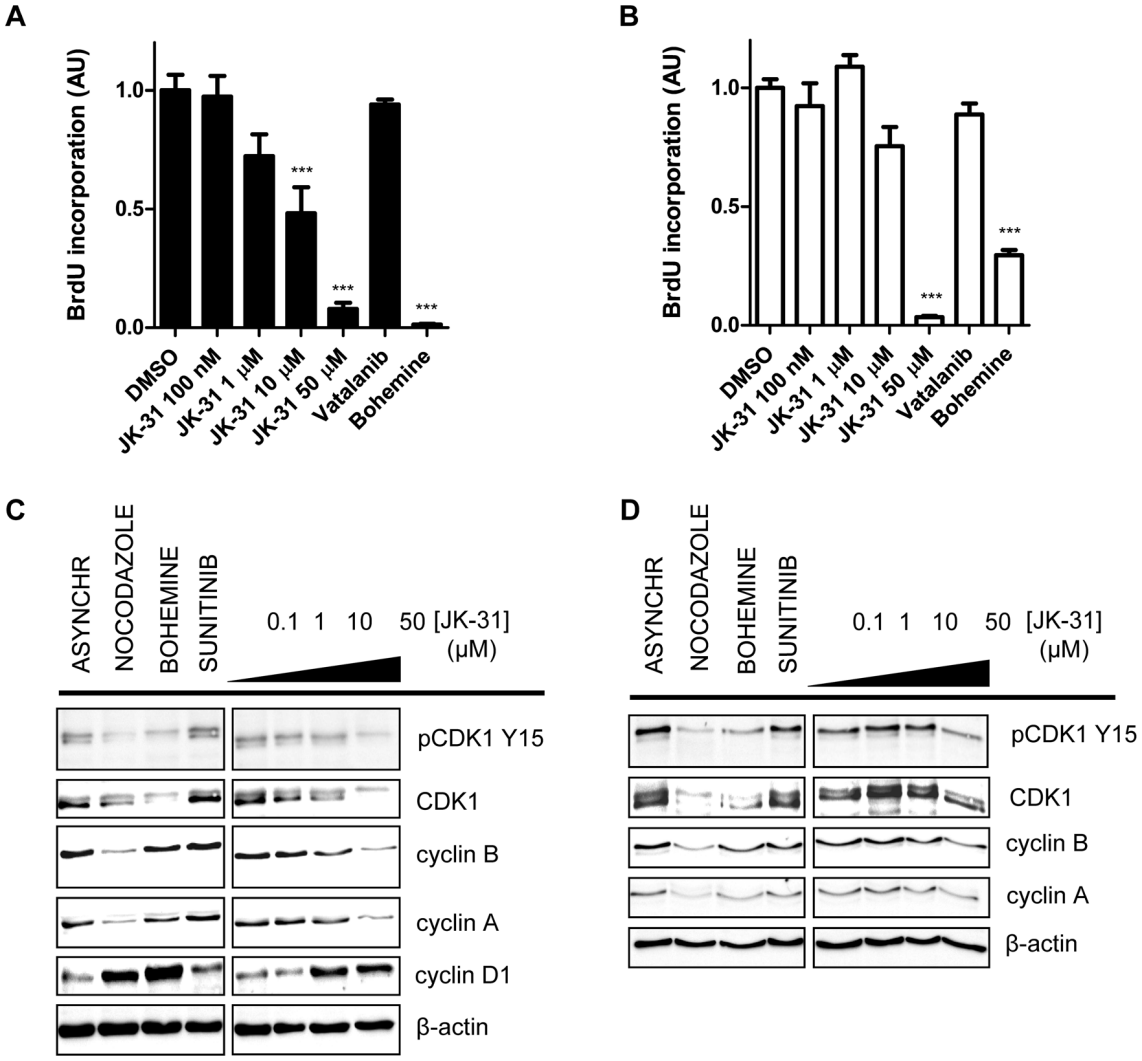
Effects of JK-31 on proliferation and cell cycle status of primary human endothelial cells and human breast cancer cells. (A) HUVECs and (B) MCF-7 cells were treated with DMSO, JK-31 (0.1, 1, 10, 50 µM), vatalanib (10 µM) or bohemine (10 µM) in full growth medium for 16 h followed by labeling with bromodeoxyuridine (BrdU; 10 µM) for 2 h and subsequent ELISA. Absorbance readings at OD_450_ were normalized and expressed relative to DMSO control. Error bars represent ± SEM (n = 4; ***p<0.001). (C) HUVECs and (D) MCF-7 cells were treated with nocodazole (200 nM), bohemine (10 µM), sunitinib (100 nM) or JK-31 (0, 0.1, 1, 10, 50 µM) for 48 h. Total cell lysates were prepared and immunoblotting carried out as described in Experimental Section. Membranes were probed for phosphorylated and total CDK1 in addition to cyclin A, B and D1 and a loading control (α-tubulin). Representative immunoblots of three independent experiments shown.

JK-31 inhibits both a key pro-angiogenic receptor tyrosine kinase, VEGFR2, and a well-described regulator of the cell cycle, CDK1. Are such effects evident in different cellular responses involving VEGFR2 and/or CDK1 that could contribute to growth of a neovascularized tumor? To address such a scenario, we first examined the effects of JK-31 on the proliferation of primary endothelial cells (Figure 5A) and the human breast cancer cell line MCF-7 (Figure 5B). Immunoblot analysis showed that both cell types express CDK1 but only endothelial cells express VEGFR2 (Figure S7A). Using a bromodeoxyuridine (BrdU) incorporation assay to monitor new DNA synthesis, we found that JK-31 inhibited proliferation of both human cell types, albeit at a higher dose range in MCF-7 breast epithelial cells (Figure 5B). A control CDK1-selective inhibitor, bohemine, showed pronounced inhibition of proliferation of both cell types; however, the VEGFR-specific inhibitor vatalanib did not significantly inhibit cell proliferation in endothelial cells (Figure 5A) or breast cancer cells (Figure 5B). A cell viability assay showed that JK-31 had no significant cytotoxic effects on endothelial cells when treated for two days at concentrations of up to 50 µM (Figure S7B).

### Effects of JK-31 on the cell cycle in both endothelial cells and breast cancer cells

VEGFR-selective inhibitors have minimal effects on endothelial cell cycle progression [18], [27], [34] whereas CDK-specific inhibitors can cause a cell cycle-specific arrest [31], [36]. Can JK-31 perturb cell cycle progression in both endothelial and non-endothelial cells? To assess this, we compared the expression and phosphorylation status of cyclins and cyclin-dependent kinases (CDKs) in endothelial cells and MCF-7 breast cancer epithelial cells treated with either JK-31, nocodazole (a cell synchronization agent), bohemine (a control CDK1 inhibitor) or sunitinib (a control VEGFR inhibitor) (Figure 5C and 5D). CDK1 is maintained in an inhibitory state by phosphorylation at residue Y15, which during transition from G_2_ to M phase is rapidly dephosphorylated to allow for entry into mitosis [31], [37]. The cell synchronization agent nocodazole disrupts microtubule polymerization, arresting cells at the G_2_/M phase boundary and leading to loss of CDK1 Y15 phosphorylation (Figure 5C and 5D; Figures S8A and S9A) [31]. In comparison to an asynchronous cell population, nocodazole also decreased levels of total CDK1, cyclin A and cyclin B, but increased cyclin D1 levels, consistent with cell cycle arrest at the G_2_/M phase boundary (Figure 5C and 5D).

Here, both JK-31 and bohemine partially mimicked the effects of nocodazole. Whereas bohemine reduced both phospho- and total CDK1 levels in both endothelial and MCF-7 cells, the effects on cyclin A and cyclin B were less pronounced (Figure 5C and 5D; Figure S8B–S8E and S9B–S9D). In endothelial cells, JK-31 showed similar effects to nocodazole at concentrations of 10 µM and above (Figure 5C; Figure S8A–S8E), whereas in MCF-7 cells, a higher JK-31 concentration was needed to elicit the same response (Figure 5D; Figure S9A-S9D), concordant with results from cell proliferation assays (Figure 5A and 5B). Sunitinib exhibited no marked effects on cell cycle protein expression or phosphorylation when compared to an asynchronous control (Figures 5C and 5D; Figure S8A–S8E and S9A–S9D). Taken together, these results suggest that JK-31 causes cell cycle arrest through inhibition of CDK1 activity (Figure 5C and 5D; Figure S8A–S8E and S9A–S9D).

**Figure 6:**
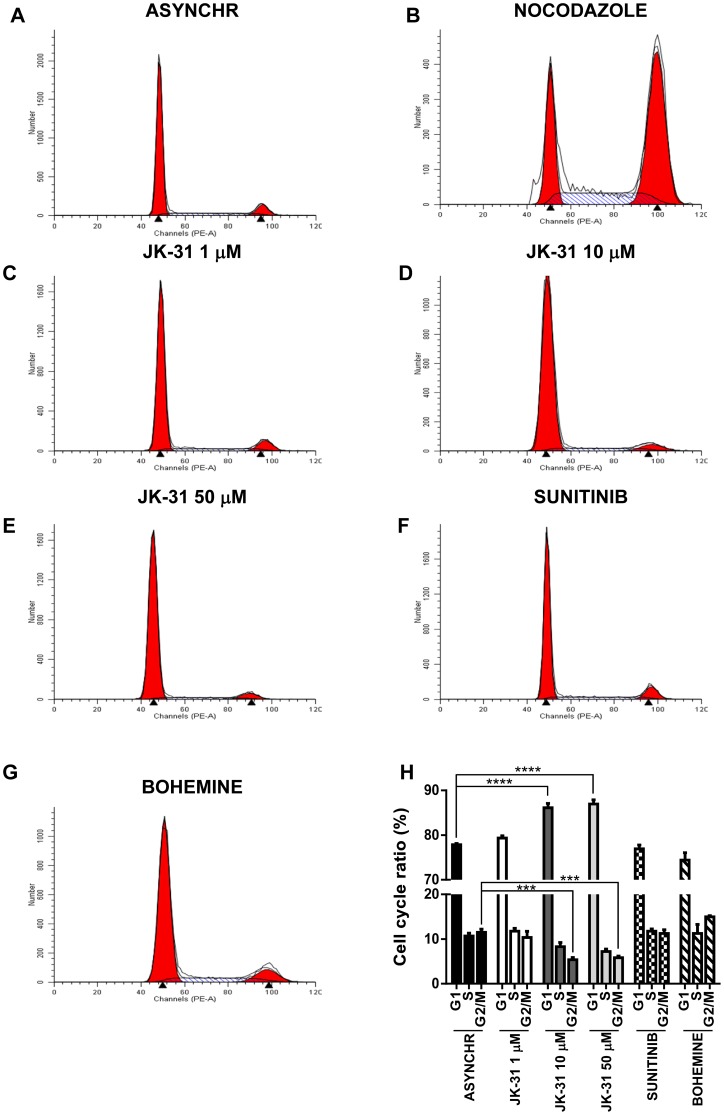
JK-31 inhibits cell cycle progression in endothelial cells. Endothelial cells were cultured in ECGM and treated with either (A) DMSO (ASYNCHR), (B) 200 nM nocodazole, (C) 1 µM JK-31, (D) 10 µM JK-31, (E) 50 µM JK-31, (F) 100 nM sunitinib or (G) 10 µM bohemine for 48 h, prior to staining with propidium iodide and assessment of DNA content using flow cytometry. (H) Quantification of cell cycle ratios after inhibitor treatment. Error bars represent ±SEM (n = 3). ***p<0.001; ****p<0.0001.

**Figure 7:**
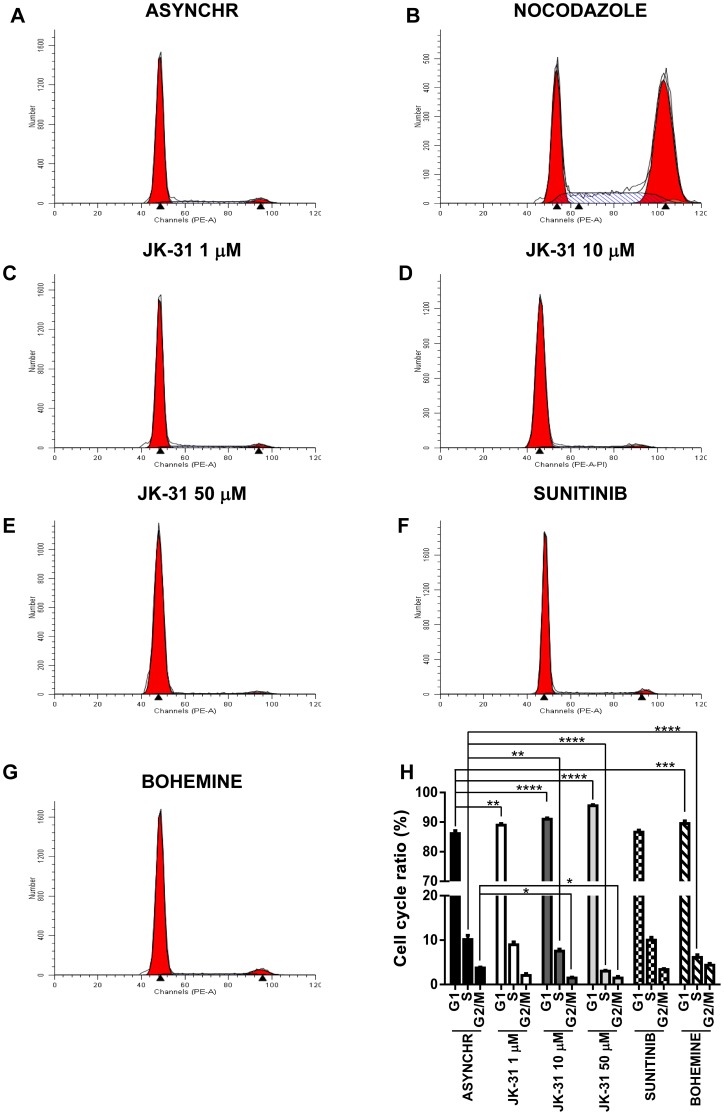
JK-31 inhibits cell cycle progression in human breast cancer cells. Human breast cancer cells were treated with either (A) DMSO (ASYNCHR), (B) 200 nM nocodazole, (C) 1 µM JK-31, (D) 10 µM JK-31, (E) 50 µM JK-31, (F) 100 nM sunitinib or (G) 10 µM bohemine for 48 h, prior to staining with propidium iodide and assessment of DNA content using flow cytometry. (H) Quantification of cell cycle ratios after inhibitor treatment. Error bars represent ±SEM (n = 3).). *p<0.05; **p<0.01; ***p<0.001; ****p<0.0001.

To investigate this further, human endothelial or breast cancer cells were treated with nocodazole, JK-31, sunitinib or bohemine for 48 h prior to fixation and analysis of cellular DNA status using flow cytometry. Cells containing n = 1 and n = 2 amount of genomic DNA were quantified (Figure 6A–7H; 7A–7H and Figure S10A–S10B), revealing that there was a significant decrease in the proportion of cells in the G2/M phase of the cell cycle in both endothelial and breast cancer cells treated with JK-31 at 10 or 50 µM, in comparison to asynchronous controls (Figure 6H and 7H). This correlated with a significant increase in the number of cells present in G1 (Figure 6H and 7H). Interestingly, there was not a significant change in the number of endothelial cells present in S phase upon JK-31 treatment (Figure 6H). Contrastingly, upon JK-31 treatment the number of breast cancer cells present in the S phase of the cell cycle was significantly decreased (Figure 7H). Treatment with nocodazole arrested both endothelial and human breast cancer cells at the G2/M phase boundary, promoting a significant shift in the cell cycle profile (Figure 6H and 7H and Figure S10A–S10B). However, sunitinib treatment had no effect on cell cycle progression vs. asynchronous controls in both cell types (Figure 6H and 7H). Upon treatment with the CDK1 inhibitor bohemine, there was a trend towards an increase in the number of endothelial and breast cancer cells present in both the S and G2/M phases of the cell cycle (Figure 6H and 7H). Interestingly, this suggested that JK-31 and bohemine inhibition of CDK1 have opposing effects on the cell cycle.

## Discussion

The clinical success of promiscuous kinase inhibitors has stimulated interest in ‘targeted polypharmacology’ i.e. the rational design of molecules that inhibit an opportune combination of kinases with optimal selectivity profiles [13]–[15], [38], [39]. There have been numerous recent examples of such ‘selectively non-selective’ kinase inhibitors. Inhibitors of multiple receptor tyrosine kinases (RTKs) (e.g. brivanib, a dual inhibitor of VEGFR and FGFR kinases) have proven effective in combating redundancy in signaling pathways [40], [41]. Type II inhibitors of Raf and platelet-derived growth factor receptor β (PDGFR-β) prevent angiogenesis by targeting endothelial cells, vascular smooth muscle cells and pericytes simultaneously [38]. When targeting tumor progression, inhibition of both lipid and protein tyrosine kinases has shown promise [14]; similarly, dual inhibition of the Aurora A and CDK1 kinases targets multiple critical stages in the cell cycle [31].

Our aim was to design a compound which inhibits two pathways involved in tumor growth. The combination of computer-aided molecular modeling strategies and kinase activity screening enabled the rapid design, pruning and validation of synthetic aminotriazole compounds, leading to the identification of JK-31, a molecule that potently inhibits both VEGFR2 and CDK1. We targeted VEGFR2 because this receptor tyrosine kinase is a known master regulator of the pro-angiogenic response [6], [42], [43]. We targeted the serine/threonine protein kinase CDK1 because it is a critical regulator of entry into mitosis, whose aberrant expression and control is exhibited in a wide range of tumors [8], [36], [37].

Our *in silico* model identified JK-31 to be an extremely potent inhibitor of both VEGFR2 and CDK1, displaying a predicted inhibition of both kinases in the mid- to low nanomolar range. This trend in potency was translated into an *in vitro* kinase assay, in keeping with our prediction that JK-31 is a more potent inhibitor of VEGFR2 than CDK1. In a cellular assay (examining effects on VEGF-A-stimulated VEGFR2 activation and downstream signaling in endothelial cells), the IC_50_ of JK-31 was approximately 10-fold higher than that observed in a cell-free phosphorylation assay using recombinant protein kinases. One possibility is that recombinant enzymes in a cell-free system are not subject to the same conformational inactivation as for the native cell-associated enzyme [38]. In contrast, many anti-angiogenic compounds display increased potency in a cellular assay than in a cell-free system relying on purified components [18], [34]. The *in vitro* potency of JK-31 is within a comparable range to an existing inhibitor, vatalanib, although the *in vivo* efficacy of JK-31 remains to be determined. However, the major advantage of JK-31 over existing anti-angiogenic therapies is a marked anti-proliferative effect, attributable to the CDK1-inhibiting properties of this compound [9], [18], [34]. We can clearly show that JK-31 has cytostatic effects on both human endothelial and breast cancer epithelial cells. In addition, JK-31 displays effects on the cell cycle consistent with arrest at the G1/S phase boundary, this effect is dissimilar to those observed with nocodazole (an inhibitor of microtubule polymerization and anti-neoplastic agent) and surprisingly, bohemine (a commercial CDK1 inhibitor). However CDK1 is known to regulate G1/S and G2/M phase transition [44], [45]. Thus, this phenomenon could be attributed to the slight differences in various cyclin expression levels between the two compounds. Whilst these effects on proliferation and the cell cycle are clear, the compound does not exhibit non-specific cellular cytotoxicity *in vitro* at the concentrations studied here. However, this does not rule out any serious side effects which could occur from using the inhibitor *in vivo*.

A previous study identified synthetic molecules which satisfy the structural constraints of both pro-angiogenic receptor tyrosine kinases and cyclin-dependent kinases; however, whilst some inhibition of cancer cell proliferation was evident, effects on angiogenesis were not shown [46]. The overall effect of JK-31 on breast cancer epithelial cells (which lack VEGFR2) was a reduction in cell proliferation. Moreover, combined inhibition of CDK1 and VEGFR2 in endothelial cells led to an overall anti-angiogenic effect: JK-31 inhibited activation of VEGFR2 and its downstream signaling pathways in VEGF-A-treated endothelial cells and simultaneously blocked cell proliferation. JK-31 also showed pronounced inhibition of FGF-stimulated signaling pathways, another common feature of many anti-angiogenic compounds [18]. We propose that inhibition of FGF-related pathways would be beneficial to overcome redundancy observed with more specific kinase inhibitors [40], [41], [47]. Moreover JK-31 does not display inhibition of either EGF- or IGF-1-stimulated signaling pathways, demonstrating the relative specificity of this compound. Potent inhibition of receptor tyrosine kinase activity and growth factor-mediated signaling translates to equally potent inhibition of VEGF-A- and bFGF-stimulated endothelial tube formation *in vitro* and angiogenic sprout formation in an *ex vivo* murine aortic ring model. In addition, JK-31 potently inhibits wound closure in an *in vitro* model.

## Conclusion

In summary, we report the development of JK-31, an inhibitor directed against two structurally dissimilar protein kinases: the VEGFR2 receptor tyrosine kinase and the CDK1 serine/threonine protein kinase. The current study argues that a rationally designed inhibitor based on comprehensive structure-function analyses can produce a synergistic effect which not only targets new blood vessel formation and development, but also cell cycle progression. These findings further realize the potential to disrupt both tumor angiogenesis and tumor growth with one compound. Current challenges to anti-angiogenic and anti-cancer therapy include the targeting of pathways that control refractoriness and multidrug resistance (e.g. by inhibitor-mediated upregulation of ABC transporters), identification of biomarkers to predict response to therapy and elucidation of inhibitors which not only retard tumor growth but also cause tumor regression [48]–[51]. Although further work is needed to fully characterize the selectivity profile of JK-31, our study serves as a benchmark for the development and refinement of similar multi-kinase inhibitors, enabling modulation of multiple cellular outputs associated with specific disease states.

## Supporting Information

Figure S1 **Validation of primary endothelial cells.** (A) Confluent HUVEC monolayers (grown as described in Materials and Methods) were visualized using phase contrast microscopy. 4X magnification; bar, 1000 µm. For immunofluorescence microscopy, confluent HUVECs were labeled with (B) anti-Von Willebrand Factor (VWF), (C) anti-VEGFR1, (D) anti-VEGFR2 and (E) anti-PECAM-1 (CD31) primary antibodies followed by secondary anti-species antibodies conjugated to AlexaFluor488 (green) and visualized using fluorescence miscroscopy. Nuclei were labeled with a DNA stain, DAPI (blue). Bar, 200 µm.
(TIF)

Figure S2 **Homology modeling of CDK1 and sequence alignments.** (A) Structural representation of sequence-aligned CDK1 and VEGFR2 kinase domains with inset showing the ATP-binding pocket. VEGFR2 crystal structure is shown in yellow. CDK1 crystal structure is shown in blue/grey. (B) Sequence alignment of CDK1 and CDK2 proteins used to create a homology model in Figure 1. Further information on the homology model and docking studies can be found in Materials and Methods.
(TIF)

Figure S3 **Validation of a CDK1 homology model.** Chemical moieties predicted to interact with residues of the CDK1 ATP-binding domain hinge region are shown in blue. (A) Chemical structure of JK-31 showing moieties predicted to interact with CDK1. (B) Chemical structure of the previously characterized CDK1 inhibitor AT7519, showing moieties predicted to interact with CDK1. (C) JK-31 and AT7519 were docked into the CDK1 homology model simultaneously using the program *Glide* to create a docking overlay. Both compounds were predicted to make hydrogen bond contacts with residues E81 and L83 of the homology model. (D) Chemical structure of the diaminothiazole inhibitor previously described [30] showing moieties predicted to interact with CDK1. (E) JK-31 and the diaminothiazole inhibtor were docked into the CDK1 homology model simultaneously using the program *Glide* to create a docking overlay. Both compounds were predicted to make hydrogen bond contacts with residues E81 and L83 of the homology model. Magenta structure  =  JK-31; yellow structure  =  AT7519; cyan structure  =  diaminothiazole inhibitor; green structure  =  residues of CDK1 kinase domain.
(TIF)

Figure S4 **Docking overlay of JK-31 and previously characterized VEGFR2 inhibitors.** Chemical moieties predicted to interact with residues of the VEGFR2 ATP-binding domain hinge region are shown in blue. (A) Chemical structure of PD173074 (a dual VEGFR/FGFR inhibtor) showing moieties predicted to interact with VEGFR2. (B) JK-31 and PD173074 were docked into the VEGFR2 kinase domain simultaneoulsy using the program *Glide* to create a docking overlay. Both compounds were predicted to make hydrogen bond contacts with residues E917 and C919 of VEGFR2. (C) Chemical structure of a derivative of the VEGFR2 inhibitor pazopanib showing moieties predicted to interact with VEGFR2. (D) JK-31 and the pazopanib derivative were docked into the VEGFR2 kinase domain simultaneously using the program *Glide* to create a docking overlay. Both compounds were predicted to make hydrogen bond contacts with residues E917 and C919 of VEGFR2. (E) Chemical structure of a derivative of the VEGFR2 inhibitor JK-P3 showing moieties predicted to interact with VEGFR2. (F) JK-31 and JK-P3 were docked into the VEGFR2 kinase domain simultaneously using the program *Glide* to create a docking overlay. Both compounds were predicted to make hydrogen bond contacts with residues E917 and C919 of VEGFR2. Magenta structure  =  JK-31; yellow structure  =  PD173074; cyan structure  =  pazopanib derivative; pink structure  =  JK-P3; green structure  =  residues of VEGFR2 kinase domain.
(TIF)

Figure S5 **Quantification of JK-31 inhibition on growth factor-stimulated endothelial cell signal transduction.** Quantification and statistical analysis of JK-31 treatment on (A) PLCγ1-pY783, (B) Akt-pS473 and (C-E) ERK1/2-pT202/Y204 levels in response to (A-C) VEGF-A, (D) aFGF or (E) bFGF stimulation of endothelial cells. Error bars represent ±SEM (n = 3). *p<0.05; **p<0.01; ***p<0.001.
(TIF)

Figure S6 **Relative specificity of JK-31 for the VEGF-A-VEGFR2 signaling pathway.** JK-31 does not inhibit (A) EGF- or (B) IGF-1-mediated signaling in endothelial cells. HUVECs were pre-treated with JK-31 (0, 0.1, 1, 10 or 50 µM) for 30 min followed by a 10 min stimulation with either (A) EGF (50 ng/ml) or (B) IGF-1 (100 ng/ml) in the presence of JK-31. Total cell lysates were prepared and processed for immunoblotting. Levels of phosphorylated Akt and ERK1/2 were analysed using phospho-specific antibodies. Membranes were stripped and re-probed for total protein levels and a loading control (α-tubulin). Representative immunoblots of three independent experiments are shown.
(TIF)

Figure S7 **JK-31 does not compromise endothelial cell viability.** (A) Relevant protein expression in HUVECs and MCF-7 cells. Total cell lysates were processed for immunoblotting. Membranes were probed with antibodies raised against VEGFR2, CDK1 and β-actin. Representative immunoblot was shown. (B) HUVECs were treated with DMSO or JK-31 (0.1, 1, 10, 50 µM) in full growth medium for 48 h followed by MTS assay and subsequent measurement of absorbance at OD_490_. Error bars represent ±SEM (n = 5).
(TIF)

Figure S8 **Quantification of the effects of JK-31 on cell cycle status in endothelial cells.** Quantification and statistical analysis of (A) phospho-CDK1, (B) CDK1, (C) Cyclin B, (D), Cyclin A and (E) Cyclin D1 levels in endothelial cells treated with nocodazole, bohemine, sutinib and JK-31 for 48 h. Statistics presented relative to ansyncronous controls. Error bars represent ±SEM (n = 3). *p<0.05; **p<0.01; ***p<0.001.
(TIF)

Figure S9 **Quantification of the effects of JK-31 on cell cycle status in human breast cancer cells.** Quantification and statistical analysis of (A) phospho-CDK1, (B) CDK1, (C) Cyclin B and (D), Cyclin A levels in human breast cancer cells treated with nocodazole, bohemine, sutinib and JK-31 for 48 h. Statistics presented relative to ansynchronous conrtrols. Error bars represent ±SEM (n = 3). *p<0.05; **p<0.01; ***p<0.001.
(TIF)

Figure S10 **Quantification of the effects of nocodazole treatment on cell cycle progression in endothelial and human breast cancer cells.** Quantification and statistical analysis of cell cycle progression in (A) endothelial (HUVEC) or (B) epithelial (MCF-7) cells upon treatment with 200 nM nocodazole for 48 h. Statistics presented relative to ansynchronous (ASYNCHR) controls. Error bars represent ±SEM (n = 3). ***p<0.001; ****p<0.0001.
(TIF)
